# 

**Published:** 1918-01

**Authors:** 


### SUBJECT INDEX TO VOLUME LXXII

### EVENT AND COMMENT

Another War Blunder, 353.

Army and Navy Dental Service, 502.

Army Medical Museum and Library, 251.

Black Historical Exhibit, 403.

Black Memorial, 5, 352.

Callahan, Dr. John R., 101.

Demobilization, 555.

Dental Metals Situation Modified, 556.

Dentistry's Contribution to the War, 552.

Dentists in the Army, 152.

Dentistry and the War, 301.

How Meet the Demand of Dentistry, 201.

Important Decisions, 351.

Meritorious Recognition, 251.

National Dental Association, 4.

National Meetings, 402.

National War Council of Y. M. C. A., 453.

Ohio State Annual Meeting, 7.

Our New Responsibilities, 554.

Preparedness League, 302.

Prosthesis for Oral Mutilations, 252.

Research Institute, 5.

Scientific Prosthetic Restorations, 151.

Standardization of State Licensing Examinations, 501.

Stenographers and Typists, 452.

The New Military Dental Course, 557.

The War is Over, 551.

Tributes to Dr. G. V. Black, 408.

Verdict in Taggart Suit, 153.

Veterans of the World's War, 555.

War and Dental Education, 451.

War's Recompense, 51.

Western Dental Journal, 3.

What Has the War Done for Dentistry, 553.

### FORMAL PAPERS

Address on Oral Hygiene. By Dr. H. Emerson, 472.

A First-Hand Picture of the Kaiser and Berlin in War Times. By Walter W. Sage, D.D.S., 377.

American Institute of Dental Teachers, 53.

Apothesine Anesthesia. By O. O. Carter, D.D.S., 559.

Bacteriology and Dental Caries, 531.

Casting Gold Inlays. By R. Ottolengui, D.D.S., 112.

Comment on Dr. Stillman's Paper. By A. M. Thornton, D.D.S., 186.

Comparison of Office Policies with Reference to Pulp Exposures. By O. E. Inglis, D.D.S., 425.

Dedication Oration of the G. V. Black Memorial. By A. W. Thornton, D.D.S., 460.

Detroit Dental Club's Clinics. By W. C. Trotter, D.D.S., 303.

Devitalized Teeth. By Joseph Novitzky, D.D.S., 310.

Devitalizing Pulps for Crowns. By H. J. Goslee, D.D.S., 268.

Diagnosis and Treatment of Traumatic Occlusion. By Clarence F. Brooks, D.D.S., 189.

Diagnosis and Treatment of Root Canals. By Edgar D. Coolidge, D.D.S., 356.

Effect of Time and Wear on the Human Teeth. By A. E. Webster, D.D.S., 204.

Efficiency Dentistry. By Alfred A. Crocker, 11.

England's Mistakes in Regard to Army Dentistry, 537.

Essentials and Non-Essentials in Dental Education. By Julio Endelman, 476.

Exact Method of Locating the Apical Foramen. By L. E. Custer, D.D.S., 420.

Extracting Teeth with Proper Elevators. By Dr. H. L. Feldman, 323.

Fighting Colds. By Dr. E. R. Petskey, 128.

Flavine, the Antiseptic, 37.

Food and Teeth, 469.

Gold Inlay Not Easily Made. By J. J. Travis, D. D. S., 439.

Importance of the Articular Cartilage of the Mandible. By R. Summa, D.D.S., 330.

Impressions of the N. D. A. Meeting. By Frank Gray, D.D.S., 74.

Indications and Practical Application of Local Anesthesia. By P. G. Puterbaugh, D.D.S., 62.

Infiltration, Conductive and Intraosseous Anesthesia. By George H. Hein, D.D.S., 126.

Ionization in the Treatment of Cicatrices about the Face, 480.

Is it Structure or Environment? By C. N. Johnson, D.D.S., 213.

Malleted Foil Fillings. By J. M. Prime, D.D.S., 440.

Medical Education in U. S., 534.

Membership Report N. D. A., 40.

Municipally Educated Dentists, 540.

Need of a National Board of Dental Registration. By M. Dewey, D.D.S., 275.

Nerve Blocking. By Arthur E. Smith, D.D.S., 14.

Oklahoma Post Graduate School, 104.

Positive and Direct Method of Casting Aluminum Plates. By G. P. Baughman, D.D.S., 235.

Preparing Root Canals for Filling. By W. G. Ebersole, D.D.S., 273.

Present Status of Dental Service in U. S. Army, and Effect of War on Dental Education. By R. Ottolengui, D.D.S., 514.

Pyorrhea Alveolaris. By Julio Endelman, 116.

Pyorrhea Alveolaris. By A. H. Merritt, D.D.S., 6.

Reading Radiographs. By F. D. Price, D.D.S., 56.

Relation of the Dental Profession to the Government, 518.

Relative Efficiency of Medicaments for Sterilizing Tooth Structure. By H. M. Brooks and W. A. Price, D.D.S., 364.

Removing Impacted Lower Third Molars. By James H. Howell, D.D.S., 115.

Removable Restorations. By Dayton D. Campbell, D.D.S., 26.

Report of Dental Education Council of America. By H. Banzhap, D.D.S., 505.

Reporting Accidents from Local Anesthetics, 133.

Restoration of Abnormal Gum Tisue. By A. C. Fones, D.D.S., 429.

Restoration of Abnormal Mouth Conditions by Surgical Treatment for Prosthesis. By J. P. Ruyl, D.D.S., 229.

Retention of Full Dentures. By R. E. Hall, D.D.S., 153.

Rochester Dental Dispensary Report, 55.

Root Infection and Systemic Disease, 524.

Root Canal Controversies. By Emery Jones, D.D.S., 41.

Save the Deciduous Teeth, 52.

Selection of Anesthetic and X-Ray in Diagnosis. By Kurt H. Thoma, D.M.D., 221.

Sanitary Care of Enlisted Men. By Antoinette Funk, 520.

Silicate Cements. By Thos. Cowling, 317.

Silicate Fillings. By E. Noyes, D.D.S., 253.

Silicate Fillings. By W. V-B. Ames, D.D.S., 259.

Simple, Yet Effective Means for Making Attachment to Teeth with Vital Pulps, for Removable Bridges and Partial Plates. By G. Walter Dittmar, 574.

Sinusless-Cronic Dento-Alveolar Abscess. By Julio Endelman, D.D.S., 334.

Some Observations on Bone Grafts. By M. M. Du Fourmentel and Frison, 354.

Some Thoughts on Mouth Hygiene. By F. H. Skinner, D.D.S., 454.

Some Vital Phases of Jaw Fracture. By C. J. Lyons, D.D.S., 368.

Studies of Focal Infections. By A. D. Black, D.D.S., 105.

Suggestion Used in the Practice of Dentistry. By Dr. R. E. Denny, 278.

Teaching Oral Hygiene to Recruits. By H. J. Brachman, D.D.S., 121.

Tendencies in Operative Ideals. By A. H. Hipple, D.D.S., 442.

The Control of Focal Infections. By C. H. Mayo, M.D., 561.

The Importance of Bacterial Findings in the Treatment of Infection. By M. LaRoche, D.D.S., 466.

Toxicity of Cocaine and Novocaine in Man. By Dr. Geo. B. Roth, 325.

Traumatism Due to Faulty Coordinating Bridge-Work. By Paul R. Stillman, D.D.S., 172.

Untenable Positions with Regard to the Problem of Immunity to Dental Caries. By H. P. Pickerill, 410.

U. S. War Savings Stamps, 136.

Value of Proximal Contacts. By F. H. Orten, D.D.S., 270.

War Emergency and Medical Education. Journal A. M. A., 583.

What Not To Do for Patients. By F. W. Sage, D.D.S., 432.

### INDENTS

Adaptation to Cavity Walls, 140.

Advantage of Gold Inlay, 445.

Advice to Dentist's Assistants, 190.

Alkalis in the Treatment of Influenza, 588.

Analine as a Germicide, 346.

Anesthetizing the Tonsils, 388.

Apothesine, 146, 147.

Apothesine in Dentistry, 286.

Army Dental Corps, Appointments, 486.

A Real Bull, 194.

Assuring the Timid, 287.

Attaching Bridges to Vital Teeth, 288.

Black Memorial, 486.

Capping Pulps, 388.

Care of Children's Teeth, 141.

Care of Handpiece, 480.

Carving Amalgam Restorations, 484.

Case of Focal Infection, 140.

Casting Watt's Metal Plates, 145.

Chloro-Percha Does Not Radiograph, 288.

Clinical Methods of Treating Hyper-Sensitive Dentine, 192.

Cigar Ash, 46.

Conductive Anesthesia, 192.

Constipation, 545.

Cotton Rolls Kept in Place, 145.

Crown and Bridge Investment, 194.

Cure for Sensitive Incisal Surfaces, 286.

Danger in Pressure Anesthesia, 449.

Dental Infection, 338.

Dental Treatments for Mothers, 345.

Dental Use for Wounded Soldiers, 244.

Dentine, 241.

Denture Polishing Material, 284.

Diagnosing Hemophilia, 242.

Difference Between Prophylactic Treatment and Prophylaxis, 385.

Efficiency of Dental Medicaments, 244.

Enough Army Dentists, 383.

Expensive Expectoration, 240.

Forsythe and the Halifax Disaster, 87.

Gorgas Points to Good Coming from War, 485.

Granuloma Advantageous, 339.

Hare-Lip and Cleft-Palate, 194.

Help for Inlay Technic, 285.

How Potato Flour is Made, 343.

Hypodermic Needles, 86.

Improvement in Dental Hygiene, 487.

Infallible Root Fillers, 447.

Infiltration Anesthesia, 196.

Interrupted General Anesthesia, 447.

Investigate Powdered Glass Reports, 240.

Is Sugar a Necessity, 144.

Jaw Breaker, 140.

Kidney Infection a Result of Pyorrhea Alveolaris, 337.

Let Your Face So Shine, 381.

Licenses Revoked, 291.

Logical Nomenclature, 47.

Lower Impressions, 141.

Manipulating Modeling Compound, 86.

Medical Fallacies, 45.

Milk for Paris Babies, 545.

Musical Solo, 144.

Mustard Oil Inhalation for Toothache, 197.

N. D. A. Research Institute, 148.

Nerve Blocking in Fractures, 145.

Nerve Blocking Injections, 386.

New Anesthetic, Apothesine, 147.

New Method of Constructing Continues Gum Denture, 337.

One Nonessential Industry, 344.

Oral Sepsis and the Digestive Apparatus, 448.

Oxide of Zinc and Oil of Cloves, 586.

Peanuts as Food, 338.

Perfect Alibi, 88.

Pioneers in Root Resection, 289.

Plaster Models, 543.

Plate Polishing, 195.

Platinum Commandeered, 243.

Polishing Vulcanite Plates, 242.

Poor Kaiser Forgot the Yam, 382.

Post Operative Pain of Extraction, 199.

Preparing Root End for Crown, 285.

Present Status of the Treatment of Syphilis, 587.

Pressure Anesthesia, 541.

Preventing Alcoholic Shock to Pulp, 198.

Procaine and Novocaine Identical, 384.

Pulp Cappers, 484.

Put Out, 45.

Quiet and Comfort, 47.

Recovering a Swallowed Denture, 243.

Removal of Gutta-Percha Root Filling, 240.

Removal of Old Root Filling, 446.

Resolutions Sent to President Wilson, 143.

Root Canal Antiseptic, 139.

Root Canal Therapeutics, 286.

Root Dentimeter, 546.

Salt Solution, 45.

Saving in Concrete Terms, 341.

Self-Reliance, 542.

Sensitive Cervical Surfaces, 484.

Services Rendered by the Canadian Dental Corps, 485.

Shoulder Crown Aid, 545.

Slow Injections, 191.

Softening Beeswax, 486.

Soldering, 240.

Soldier Dental Mechanics, 284.

Sterilizing Anesthetic Solutions, 387.

Sterilizing the Dental Office, 342.

Sterilizing Dentine, 242.

Sterilizing Hand Brushes, 88.

Sterilizing the Apical Area, 241.

Sterilizing Operative Field, 87.

Sweating Feet, 199.

Teeth Do Not Control Mandibular Movements, 191.

Ten Years, 195.

The Telephone in Surgery, 592.

To an Old Tooth, 86.

To Avoid Air Bubbles, 285.

To Desensitize Teeth for Scaling, 243.

To Lessen Pain, 45.

To Refit Vulcanite Denture without Revulcanizing, 449.

To Renew Arkansas Stones, 342.

Toothache Preventable, 143.

Ulcerative Stomatitis, 589.

Uncle Sam's Boys, 591.

Uniformed Dentists' Assistants, 590.

U. S. Dental School, 46.

Use of Saccharin, 544.

Ventilation in Schools, 445.

Vinegar for Softening Plaster, 287.

Violet Ray, 196.

Vulcanite Repair, 586.

War and Dental Supply Business, 197.

War Substitutes, 341.

When to Use Pressure Anesthesia, 543.

What the Expert Anesthetist Should Be, 590.

Widening the Arch to Facilitate Breathing, 246.

Zinc Dies and Counter-Dies, 242.

BIBLIOGRAPHY

A Child's Book of the Teeth, 450.

Dental Electro-Therapeutics, 299.

Electrolytic Medication, 299.

Elementary Dental Radiography, 300.

General Pathology and Bacteriology for Dental Students, 298.

Modern Dentistry, 49.

Modern Dentistry for the Laity and Industrial Dentistry, 450.

Oral Diseases and Malformations, 499.

OBITUARY

Edward C. Harmeyer, 50.

J. R. Callahan, 101.

ANNOUNCEMENTS

Army Dental Corps Legislation, 99.

American Institute of Dental Teachers, 48, 148, 592.

American Society of Orthodontists, 247, 347.

Clinics of N. D. A., 293.

Connecticut Dental Society, 48, 89.

Dental Protective Association, 592.

Forsythe's New Editor, 48.

Forsythe Infirmary Appointments, 292.

Illinois State Society, 200.

Indiana State Society, 200.

Iowa Examining Board, 199, 390.

Kentucky Dental Association, 89, 247.

Michigan Examiners, 194, 247.

Motor Cars for Cantonments, 497.

National Association of Examiners, 247, 347.

National Dental Association, 294, 347.

National Association Dental Faculties, 200, 347.

Navy Dental Legislation, 96.

New Naval Legislation, 393.

New York Dental Society, 200.

Ohio State Dental Society, 546.

Preparedness League, 92, 248, 295, 397, 490.

Preparedness League Notes, 546.

Professional Appeal, 89.

Red Cross Ambulance, 548.

Red Cross Dental Motor Car, 547.

Texas Dental Society, 48, 89.

U. S. Employment Service, 390.

Vacancies in Dental Corps, 93, 488.

West Virginia State Association, 89.

West Virginia State Board of Examiners, 247.

BIOGRAPHIC

Almon, H., 241.

Amenta, J. V., 146.

Ames, W. V-B., 259.

Ash, Charles, 88.

Beach, J. W., 548.

Banzhaf, H. L., 505.

Baughman, G. P., 235.

Black, A. D., 105.

Bliler, H. E., 45.

Brachman, H. J., 121.

Brooks, M. M., 364.

Brooks, C. F., 187.

Buckley, J. P., 446.

Burke, George F., 487.

Callahan, J. R., 101.

Campbell, D. D., 26.

Carter, O. O., 559.

Chandler, J. V., 87.

Coolidge, E. D., 356.

Cormany, J. W., 286.

Crocker, A. A., 11.

Crockett, F. S., 338.

Custer, L. E., 420, 449.

Cross, A. H., 199, 544.

Cross, H. D., 87.

Cowling, Thomas, 317.

Davis, W. C., 289.

Denny, Dr., 278.

De Vries, A., 242, 243.

Dewey, M., 275.

Dittmar, G. W., 574.

Du Fourmentel, 354.

Dungan, F. L., 484.

Ebersole, W. G., 241, 242, 273, 287.

Ely, Thomas C., 588.

Emerson, H., 472.

Endelman, Julio, 116, 334, 476, 542, 589.

Federspiel, Dr., 291, 341.

Feldman, H. M., 323.

Fones, A. C., 429.

Frahm, F. W., 192, 194, 195, 198, 242, 285, 484, 545, 586.

Frison, Dr., 354.

Funk, Antoinette, 54, 520.

Goslee, H. J., 268.

Gray, B. F., 74.

Grove, C. J., 140.

Hall, Rupert E., 141, 153.

Hipple, A. H., 442.

Hein, G. H., 126.

Hoover, H., 342.

Howell, James H., 115.

Inglis, Otto E., 425.

Johnson, C. N., 52, 191, 213.

Jones, Emery, 41.

La Roche, M., 466.

Lee, Dr., 47.

Light, R. E., 45.

Lucas, Carl, 142.

Lyons, C. J., 368.

Mayo, C. H., 561.

McClarty, Dr., 484.

McCormick, A. S., 590.

McManus, J. F., 240.

Merritt, A. H., 6.

Morris, H. J., 243.

Novitzky, J., 310.

Noyes, Edmund, 253.

Nyman, J. E., 87, 192.

Orten, F. H., 270.

Ottolengui, R., 112, 390, 514.

Petskey, E. R., 128.

Pickerill, H. P., 410.

Price, F. D., 56.

Price, H., 546.

Price, W. A., 150, 364.

Prime, J. M., 141, 440.

Puterbaugh, G. P., 62, 197, 342.

Roper, H. R., 143.

Roth, Dr. G. B., 325.

Roubert, L. N., 285, 287.

Roach, F. E., 288.

Ruyl, J. P., 229.

Sage, F. W., 432.

Sage, W. W., 377.

Scheiman, P., 541.

Schamberg, Jay F., 587.

Shaw, D. M., 486.

Sitherwood, G. B., 347.

Skinner, F. H., 454.

Smith, Arthur E., 14, 145, 191, 387, 388.

Smith, Roy L., 542.

Starr, A. R., 447.

Stillman, P. R., 172.

Summa, R., 330.

Thoma, K. H., 221.

Thornton, A. W., 186, 460.

Travis, J. J., 439.

Trotter, W. C., 303.

Tumulty, J. P., 144.

Ulsaver, E. S., 286, 587.

Wadleigh, W. M., 45, 140.

Walls, Dr., 445.

Weber, R. M., 86.

Webster, A. E., 204.

### ANNOUNCEMENTS

CONNECTICUT STATE DENTAL SOCIETY. The fifty-fourth annual meeting of the Connecticut State Dental Association will be held at the Hotel Taft, New Haven, Connecticut, April 18, 19 and 20, 1918.

Mystic, Conn. G. S. B. LEONARD, *Secy.*

AMERICAN INSTITUTE OF DENTAL TEACHERS. The next annual meeting of the American Institute of Dental Teachers will be held at Hotel Schenley, Pittsburgh, Pennsylvania, January 29, 30, 31, 1918.

The meeting as usual will be devoted to dental teaching—a number of the papers will deal with situations arising from war conditions. A cordial invitation is extended to all interested in dental teaching.

ARBAM HOFFMAN, *Secy.*

381 Linwood Ave., Buffalo, N. Y.

ANNUAL CONVENTION OF THE TEXAS STATE DENTAL SOCIETY. The thirty-eighth annual convention of the Texas State Dental Society will be held at San Antonio, Texas, the famous City of the Alamo, April 10, 11 and 12, 1918. Members of other state societies are cordially invited to attend. J. G. FIFE, *Secy.*

736 Wilson Building, Dallas, Texas.

EDITOR FOR FORSYTHE DENTAL INFIRMARY. Dr. Walter A. Bradford has been appointed editor of all transactions in this institution. The idea is to preserve and file or publish all statistical and other material that may be of historic or scientific value which may develop in the work of this institution. If accurately done it should be of great value.

